# Theory-Informed Design of a Tailored Strategy for Implementing Household TB Contact Investigation in Uganda

**DOI:** 10.3389/fpubh.2022.837211

**Published:** 2022-03-24

**Authors:** J. Lucian Davis, Irene Ayakaka, Joseph M. Ggita, Emmanuel Ochom, Diana Babirye, Patricia Turimumahoro, Amanda J. Gupta, Frank R. Mugabe, Mari Armstrong-Hough, Adithya Cattamanchi, Achilles Katamba

**Affiliations:** ^1^Department of Epidemiology of Microbial Diseases, Yale School of Public Health, New Haven, CT, United States; ^2^Center for Methods in Implementation and Prevention Science, Yale School of Public Health, New Haven, CT, United States; ^3^Pulmonary, Critical Care, and Sleep Medicine Section, Yale School of Medicine, New Haven, CT, United States; ^4^Uganda Tuberculosis Implementation Research Consortium, College of Health Sciences, Makerere University, Kampala, Uganda; ^5^Liverpool School of Tropical Medicine, Liverpool School of Tropical Medicine (LSTM) International Multidisciplinary Programme to Address Lung Health and TB in Africa (IMPALA) Program, Liverpool, United Kingdom; ^6^Uganda Ministry of Health, Kampala, Uganda; ^7^Department of Social and Behavioral Sciences, School of Global Public Health, New York University, New York, NY, United States; ^8^Department of Epidemiology, School of Global Public Health, New York University, New York, NY, United States; ^9^Division of Pulmonary, Critical Care, Allergy, and Sleep Medicine, San Francisco General Hospital, University of California, San Francisco, San Francisco, CA, United States; ^10^Curry International Tuberculosis Center, University of California, San Francisco, San Francisco, CA, United States; ^11^Division of Epidemiology and Biostatistics, University of California, San Francisco, San Francisco, CA, United States; ^12^Clinical Epidemiology Unit, Department of Medicine, School of Medicine, College of Health Sciences, Makerere University, Kampala, Uganda

**Keywords:** implementation strategies, implementation science, intervention design, tuberculosis, Uganda, low-and-middle-income countries, implementation mapping, contact investigation

## Abstract

Since 2012, the World Health Organization has recommended household contact investigation as an evidence-based intervention to find and treat individuals with active tuberculosis (TB), the most common infectious cause of death worldwide after COVID-19. Unfortunately, uptake of this recommendation has been suboptimal in low- and middle-income countries, where the majority of affected individuals reside, and little is known about how to effectively deliver this service. Therefore, we undertook a systematic process to design a novel, theory-informed implementation strategy to promote uptake of contact investigation in Uganda, using the COM-B (Capability-Opportunity-Motivation-Behavior) model and the Behavior Change Wheel (BCW) framework. We systematically engaged national, clinic-, and community-based stakeholders and collectively re-examined the results of our own formative, parallel mixed-methods studies. We identified three core behaviors within contact investigation that we wished to change, and multiple antecedents (i.e., barriers and facilitators) of those behaviors. The BCW framework helped identify multiple intervention functions targeted to these antecedents, as well as several policies that could potentially enhance the effectiveness of those interventions. Finally, we identified multiple behavior change techniques and policies that we incorporated into a multi-component implementation strategy, which we compared to usual care in a household cluster-randomized trial. We introduced some components in both arms, including those designed to facilitate initial uptake of contact investigation, with improvement relative to historical controls. Other components that we introduced to facilitate completion of TB evaluation—home-based TB-HIV evaluation and follow-up text messaging—returned negative results due to implementation failures. In summary, the Behavior Change Wheel framework provided a feasible and transparent approach to designing a theory-informed implementation strategy. Future studies should explore the use of experimental methods such as micro-randomized trials to identify the most active components of implementation strategies, as well as more creative and entrepreneurial methods such as human-centered design to better adapt the forms and fit of implementation strategies to end users.

## Introduction

More than 10 million patients develop active tuberculosis annually, but over three million are never diagnosed because they cannot or do not access diagnostic evaluation and treatment services (1). The WHO End TB Strategy, endorsed by the World Health Assembly in 2015, has called for expanding beyond “passive” facility-based diagnostic strategies to include “active” community-based approaches to finding missing individuals with undiagnosed TB (2). The archetypal example of active case finding is household TB contact investigation, an evidence-based intervention in which TB symptom screening; clinical and laboratory-based TB diagnostic evaluation; treatment for active TB disease; and preventive treatment for latent TB infection are offered to household members of newly diagnosed TB patients. Household TB contact investigation has been endorsed by WHO for routine implementation in high TB-burden countries (3, 4) based on a few high-quality studies (5–7) and a comprehensive systematic review (8). However, implementation studies suggest that the yield of contact investigation is often limited by low rates of uptake and follow-up among community members (9). Although formative research has identified explanations for poor uptake and completion, including a lack of TB-specific knowledge, fear, social stigma, dissatisfaction with clinic services, and lack of money or time to travel to clinics for evaluation (10), little has been published about what might be done to overcome these barriers and improve uptake and delivery of TB contact investigation.

Implementation strategies are specific techniques used to promote adoption, uptake, implementation, and sustainability of innovations and evidence-based practices previously known or believed to improve individual or public health outcomes (11, 12). A variety of approaches to cataloging, developing, or selecting these strategies have been proposed, including employing evidence-based implementation strategies (13) and applying behavioral theory and stakeholder engagement to design strategies targeted to intervention barriers and facilitators (14). The latter approach has much in common with implementation mapping (15), a process to develop strategies to promote adoption and implementation outcomes that is the focus of this Special Issue. The main difference is that implementation mapping is nested within a broader approach to planning and delivering multi-level health promotion activities called intervention mapping (16, 17), which includes separate procedures for designing and adapting interventions. In contrast, behavior-change theories consider client and implementer behaviors and behavior change objectives at the same time, allowing interventions and implementation strategies to be developed concurrently using the same process rather than sequentially. Given the variety of approaches, there is a critical need for case studies describing the feasibility and results of different methods for designing and selecting implementation strategies. This is especially true in low-income countries, where there is a large body of literature on effective implementation strategies targeting healthcare workers and healthcare recipients but little information about how to select among them (18).

Therefore, beginning in 2014, we undertook a series of formative and implementation studies in Uganda, a low-income country preparing to roll-out household TB contact investigation as a routine service. We first characterized factors that might prevent or enable uptake and completion of contact investigation (19) and then developed a multi-component implementation strategy to target these barriers and facilitators. We drew on published guidelines for developing complex interventions (20) and applied a systematic approach to implementation design based on a general theory of behavior change (21). Using this implementation strategy, we introduced the adapted contact investigation intervention in seven government-run primary health clinics and their surrounding communities in Kampala, Uganda, and evaluated its reach, effectiveness, fidelity/adaptation, and impact in a cluster-randomized, controlled trial (22). Here, we present a case study describing the collaborative, stakeholder-engaged process that we undertook to design and introduce our multi-component, theory-informed implementation strategy for household TB contact investigation, including the outcomes of implementation. We conclude by summarizing learnings from this experience and comparing our approach to alternative approaches including implementation mapping.

## Materials and Methods

### Project Setting and Objectives

The World Health Organization has designated Uganda one of 30 high HIV-TB burden countries (23), with an estimated TB incidence of 201/100,000 people and an estimated adult HIV prevalence of 6.5% in 2016 (24, 25). The Uganda Ministry of Health provides diagnostic evaluation and treatment services for TB and for HIV free of charge in government-run primary health centers located in every district of the country. Nevertheless, based on data provided by the Uganda National TB and Leprosy Programme, WHO has estimated that about one-third of all individuals with active TB disease in Uganda go undiagnosed and unreported to public health authorities each year (26). In 2014, this large gap in TB case notifications led Uganda to begin making plans to implement household TB contact investigation in the capital city of Kampala, the district with the country's highest TB burden.

The overall objective of this project was to adapt household TB contact investigation to the local context and design a theory-informed implementation strategy (27) to overcome barriers to delivery of this evidence-based intervention (28). Drawing on our previous formative research (19), we conceptualized contact investigation as a series of activities requiring specific behaviors involving household members and lay health workers. We sought to identify a package of components that could facilitate these activities, including (1) index patients agreeing to TB contact investigation; (2) eligible household contacts accepting screening during the home visit; and (3) household contacts with TB symptoms or predisposing factors completing TB evaluation and if diagnosed initiating TB treatment. In addition, we sought implementation components that could maximize the quality of TB contact investigation service outcomes, including safety, timeliness, effectiveness, efficiency, equity, and client-centeredness (29).

### Rationale for Using a Theory-Informed Approach to Design the Implementation Strategy

The British Medical Research Council (MRC) defines complex interventions as ones that (1) include multiple, interacting components; (2) address multiple behavioral targets among those delivering and/or those receiving the intervention; (3) target multiple groups or organizational levels; (4) address multiple outcomes that may vary between groups and cluster at different levels of an organization; and (5) allow adaptation of the intervention to local circumstances (20). Complex interventions should be designed with a sound theoretical understanding of the mechanisms through which change can be effected, a process that requires formative research (30). Moreover, a growing literature suggests that implementation strategies designed using behavioral theory are more effective than those designed without the use of theory (31, 32). Of note, the MRC guidelines do not differentiate between components targeting implementers and those that target recipients.

### Selection of an Implementation Framework

While a number of implementation frameworks are available to guide planning and introduction of this evidence-based intervention (33–35), we selected the Behavior Change Wheel (BCW) Framework for several reasons. First, it provides a taxonomy for characterizing barriers to and facilitators of evidence-based practices that is systematic and grounded in a unifying theory of behavior, the Capability, Opportunity, Motivation–Behavior (COM-B) model (21, 36). Both COM-B and an earlier, more expansive version of the model called the Theoretical Domains Framework (TDF) (14, 37)—were developed through a structured process in which experts from diverse disciplines in the social sciences and in public health systematically reviewed 19 widely used frameworks for designing behavior change interventions to identify commonalities. Their goal was to develop a single, comprehensive, and internally coherent model for understanding human behavior. The final result was a simplified theoretical model (COM-B) comprising six fundamental and overarching determinants of behavior, with the 14 component domains of the TDF nested within (and listed here in parentheses). These were *psychological capability* (knowledge; cognitive and interpersonal skills; memory, attention, and decision processes; behavioral regulation) and *physical capability* (physical skills); *physical opportunity* (environmental context and resources) and *social opportunity* (social influences); and *automatic motivation* (emotion, reinforcement) and *reflective motivation* (beliefs about capabilities; beliefs about consequences; optimism; intentions; goals) (21, 37). A second reason that we chose the BCW Framework is that it includes a systematic and comprehensive approach to identifying components of an implementation strategy, involving “intervention functions” and “behavior change techniques” that map to COM-B (or TDF) determinants of behavior using published matrices (36). The process is structured to ensure functional integrity of implementation components—the intervention function of education is suitable for deficits of psychological capability but not for those of reflective motivation, while the intervention function of incentivization is suitable for barriers of reflective motivation but not for barriers of psychological capability. The BCW framework also offers flexibility to adapt to local context and stakeholder preferences, by offering different forms through which selected intervention functions can be achieved (38). For example, the Behavior Change Techniques Taxonomy offers 15 different practical applications for delivery of the education intervention function, as well as 27 practical applications associated with the incentivization intervention function (39). Our third and final reason for selecting the BCW framework is that the simple and practical, step-wise process model prescribed by BCW was familiar to our design team, a diverse group of physicians, epidemiologists, public health practitioners, and front-line care providers working in Uganda and, at the time of this project, new to implementation science.

### Study Procedures

Like other approaches to selecting implementation strategies (13, 15, 16) and consistent with MRC guidelines on complex interventions, the BCW includes a process model to guide planning (20). Specifically, the BCW calls for implementers to follow several fundamental steps: (1) understand the behaviors by defining the implementation problem in behavioral terms, selecting at least one target behavior, specifying the core characteristics of that behavior, and identifying what needs to change; (2) identify possible implementation components by specifying the *intervention* functions (i.e., mechanisms) through which the target behaviors that need to change can be modified and the policies that could support the intervention functions at the organizational and/or societal level; and (3) identify intervention content and implementation options by selecting specific behavior change techniques, policies, and modes of delivery (36). In the Results section below, we provide the details of how we approached each of these steps in a logical progression, although in practice we sometimes diverged from this temporal sequence for convenience, since the qualitative and quantitative formative analyses were carried out in parallel under the leadership of two different team members (IA, MAH). Finally, we used a logic model to conceptualize the process of designing an individual and organizational behavior-change intervention within the larger context of an implementation strategy. Specifically, we sought to summarize the many external human and material resources that the project drew on, the extensive planning activities that were undertaken with stakeholders, and the jointly prepared outputs that influenced implementation outcomes and impact assessment.

### Human Subjects

The study protocol was approved by the Uganda National Council for Science and Technology, the Makerere University School of Medicine Research Ethics Committee, the Committee on Human Research at the University of California San Francisco, and the Yale University Human Investigation Committee.

## Results

### Step 1: Understand the Behaviors

In October, 2013, members of the research team (AC, JLD, AK) including the Uganda National TB Programme Manager (FRM) attended an international workshop to review newly issued WHO guidelines on TB contact investigation (3) and to define the target behaviors. We identified and specified three key activities requiring specific individual behaviors of health care workers (including lay health workers), index TB patients, and household TB contacts: (1) *index patients* agreeing to a home visit by lay health workers to identify household TB contacts; (2) *lay health workers* screening household contacts for TB, including referring contacts screening positive for possible active TB disease based on symptoms or predisposing factors to attend clinics for testing and evaluation; and (3) *contacts* screening positive attending clinics to complete TB evaluation and treatment by health care workers (Table 1). To better characterize these behaviors, including what might need to change, the likelihood of change, the expected spillover (i.e., indirect) effects of change, and the ease of measuring change (36), we carried out several formative assessments. The first was a qualitative study carried out between February and November 2014 in which we conducted focus group discussions with each of three of the key stakeholder groups (health care workers, lay health workers, household contacts of index TB patients) while the Uganda National TB and Leprosy Programme (NTLP) was introducing TB contact investigation in Kampala. We sought to understand their expectations about the delivery and processes of contact investigation, and to characterize barriers and facilitators of the most important behaviors using the COM-B model, as previously described (19).

**Table 1:** Specification of the behaviors required for delivery of household TB contact investigation.

**Specification domain**	**Contact investigation behaviors**		
	**Agree to contact investigation**	**Screen contacts for TB**	**Complete TB evaluation**
*Who* needs to perform the behavior?	Index TB patients	Lay health workers	Household contacts
*With whom* do they need to do it?	Health workers	Household TB contacts	With other household TB contacts who require TB evaluation or by themselves
*What* do they need to do?	Agree to contact investigation and schedule a home visit for TB screening of household contacts	Interview contacts about TB symptoms and predisposing factors for TB	Complete TB diagnostic evaluation and initiate treatment for TB if TB is confirmed
*When* do they need to do it?	As soon as possible after TB diagnosis	When one or more contacts are available	As soon as possible when the services are available
*Where* do they need to do it?	At the clinic or by phone	In the home or possibly by phone	At the clinic or wherever testing is offered
*How often* do they need to do it?	Once	Once	Regularly until TB diagnostic evaluation is complete

*TB, tuberculosis*.

*The table specifies the characteristics of each of the required behaviors in contact investigation*.

Second, we reviewed existing national and international guidelines on TB contact investigation. Uganda National TB Program guidelines specified which index TB patients should be offered contact investigation but did not provide details about how the services should be delivered (40). International guidelines went further, identifying priority populations and procedures for investigating contacts, but did not reference any published evidence on implementation procedures (3). The following year, recommendations from international experts on adaptation and implementation of TB contact investigation guidelines to local setting were released, along with standardized evaluation metrics (41, 42), and we incorporated these into our evaluation plan.

Third, we carried out and analyzed focus group discussions with health care workers, focus group discussions with lay health workers (LHWs), and interviews and one focus group with household contacts. We used the COM-B model to categorize emergent themes to identify antecedents of the specified behaviors that we could target for change (19). A full list of factors preventing each of the three key contact investigation behaviors from occurring are provided in Table 2. The most prominent of these were a lack of knowledge about TB among index patients and contacts (psychological capability) and a lack of belief in the value of engaging in TB screening and evaluation (*reflective motivation*); a lack of time and space in clinics for LHWs and index patients to meet for counseling and high travel costs to and from households for LHWs and contacts (physical opportunity); a perceived need for permission from the head of household for index patients to consent to a home visit and for contacts to attend clinic visits (social opportunity); anticipated TB-related stigma reported by household contacts, lay health workers, and health care workers and a lack of trust between clinic-based health care workers and household members (*automatic motivation*), including both index patients and contacts. The most important enabling factors noted by both clinic health workers and household contacts were the personalized and supportive services provided by LHWs.

**Table 2:** Behavioral determinants influencing adoption of three core behaviors of household TB contact investigation, and possible intervention functions specified by the behavior change wheel framework.

**COM-B determinants of behavior**	**Is change needed for the key behaviors to occur?**		
	**Agree to contact investigation (Index cases)**	**Screen contacts for TB (Lay health workers)**	**Complete TB evaluation (Contacts)**
Physical capability	No, index patients know how to agree to contact investigation.	Yes, lay health workers lack skills to elicit TB symptoms from contacts during TB screening.	No, most contacts already have the strength and skills to do this.
Psychological capability	*Yes, index patients lack knowledge about TB to understand the need for contact tracing*.	No, lay health workers know how to carry out home visits for screening.	*Yes, some contacts cannot remember to follow-up in clinic and do not understand the risk of TB*.
Physical opportunity	*Yes, clinics lack space for private conversations between index patients and household contacts*.	*Yes, lay health workers are not able to find every household contact in the home at the time of the visit(s)*.	*Yes, some contacts lack the time and money to travel to clinic*.
Social opportunity	*Yes, some index patients feel that they lack authority to consent to contact investigation, especially if not the head of household*.	No, clinic workers already trust and encourage lay health workers to perform many TB evaluation activities.	*Yes, some contacts need permission from family members to go to clinic*.
Reflective motivation	*Yes, some index patients do not believe that it is necessary or beneficial to contacts undergo TB screening and evaluation*.	No, lay health workers already believe they can and should play this role.	*Yes, some contacts do not wish to follow-up in clinic because they do not believe that it is necessary or valuable*.
Automatic motivation	*Yes, some index patients fear stigma from the household or community if a health worker visits the home for contact investigation*.	*Yes, some lay health workers are afraid of contracting TB*.	*Yes, some contacts are afraid to go the clinic and do not trust health workers*.
**Intervention functions**	*Education, Persuasion, Modeling, Environmental restructuring, Enablement*.	*Education, Training, Persuasion, Environmental restructuring, Enablement, Incentivization*.	*Education, Training, Persuasion, Environmental restructuring, Enablement, Incentivization*.

*COM-B, Capability, Opportunity, and Motivation determinants of Behavior framework; TB, tuberculosis*.

*For each of the three required behaviors for TB contact investigation, the table presents answers to the question, “Is change needed for the key behaviors to occur?” We provided answers to this question considering each of the six theoretical determinants of behavior specified by the COM-B model, drawing on focus group discussions with and/or direct observation of the core participants in contact investigation, who include lay health workers, index patients, and contacts. Finally, the list of all intervention functions appropriate to the identified COM-B determinants are drawn from published matrixes that list all intervention functions that might fit the identified determinants (36)*.

Fourth, we carried out a quantitative evaluation of the three required behaviors of household TB contact investigation in routine practice, in order to localize bottlenecks in the delivery process, as previously described (28). We found that lay health workers succeeded in scheduling the initial household visit for only 61% of index patients, and visited just 31% of index patient households. Once at the household, lay health workers screened 89% of contacts, but only 20% of contacts who screened positive subsequently attended the recommended TB evaluation visit at the clinic. In total, the conditional probability of an undiagnosed TB patient being screened and diagnosed with active TB among household contacts and linked to care was only 5% (i.e., 20% of all contacts referred, out of 89% of all contacts screened, out of 31% of all households visited).

At the conclusion of Step 1, we summarized the perspectives and experiences of stakeholders and discussed them with implementing partners. Together, we agreed that all three component behaviors could be targets for improvement during implementation, because they shared common behavioral determinants (especially barriers related to psychological capability, social opportunity, and automatic motivation); because implementation components targeting these determinants could all be delivered by lay health workers; and because the close linkage between key screening and evaluation processes within the contact investigation cascade increases the possibility of positive spillover effects on other related behaviors.

### Step 2: Identify Implementation Options

In August 2015, the implementation research team met to discuss and select the functional components of the implementation strategy using the Behavior Change Wheel framework (Table 2). To target the determinants of the first behavior, index patients agreeing to a home visit, we identified education, persuasion, and modeling as potential intervention functions best targeted to the identified behavioral determinants. Specifically, we chose education targeting psychological capability (*e.g.*, lack of knowledge of TB and benefits of screening), and persuasion and modeling targeting social opportunity (*e.g.*, lack of authority to agree to home visit), reflective motivation (*e.g.*, beliefs about consequences of exposure to a TB patient), and automatic motivation (*e.g.*, anticipated stigma). We also identified environmental restructuring (*i.e.*, changing the location of screening) and enablement (*i.e.*, social and material support from lay health workers) as intervention functions addressing the physical opportunity (*e.g.*, lack of time and private space in clinics) and other automatic motivation (*e.g.*, distrust of clinic-based health care workers) barriers.

To target the determinants of the second behavior, lay health workers screening household contacts for TB, we identified education, training, persuasion, environmental restructuring, enablement, and incentivization as possible intervention functions. Specifically, we found the most promising of these were education and training to address physical capability (*e.g.*, lack of skills in screening for TB), environmental restructuring through re-timing of visits to weekends to address physical opportunity (*e.g.*, difficulty finding every household contact at home), and persuasion to address automatic motivation (*e.g.*, fear of contracting TB in the household).

To target the determinants of the third behavior, eligible contacts completing TB evaluation clinic, we identified the same set of intervention functions—education, training, persuasion, environmental restructuring, enablement, and incentivization. The most promising of these implementation components included education to address psychological capability (*e*.*g.*, inability to remember follow-up appointments), environmental restructuring by initiating the TB testing process at home in order to address physical opportunity (*e.g*., lack time and money travel to clinic), enablement to address social opportunity (*e.g*., lack of authority to consent to home visit), and education and persuasion to address reflective motivation (*e.g*., belief of contacts that TB evaluation is not important).

### Step 3: Identify Implementation Strategy Content and Delivery Options

Having identified possible intervention functions, we proceeded to select specific behavior change techniques from the Behavior Change Techniques Taxonomy (39), design setting-specific content, and choose modes of delivery, as shown in Table 3. To convince index patients to agree to contact investigation, the first target activity, we identified multiple behavior change techniques, including (1) *providing information about health consequences* of TB/HIV; (2) ensuring that health information provided to index patients has been approved and validated by a *credible source*, the national TB program; (3) describing *anticipated regret* and possible social and environmental consequences in the form of blame by family members for not referring household contacts for evaluation; (4) providing *information about the social & environmental consequences* of not agreeing to a home visit, including putting household contacts at risk; and (5) eliciting *comparative imaginings of future outcomes* of doing and not doing the behavior. We also considered several other behavior change techniques but did not adopt them routinely because clinic-level stakeholders found them infeasible or inappropriate: (6) inviting a former index TB patient to share the difficult decision to agree to household contact investigation as a *demonstration of the behavior*; (7) *restructuring the social environment* by phoning the head of household to obtain permission for a household visit rather than asking an index patient who is not head of household to consent; and (8) *restructuring the physical environment* by screening the index patient by phone to allow greater privacy and convenience.

**Table 3:** Selected behavior change techniques, setting-specific intervention content, and modes of delivery for each of the target behaviors.

**Intervention function**	**Behavior change technique**	**Setting-specific intervention content**	**Mode of delivery**	**Implement?**
**Agree to contact investigation (index cases)**				
Education	Information about health consequences	See examples below under “Complete TB evaluation.”	Lay health worker	Yes
Persuasion	Credible source	See examples below under “Complete TB evaluation.”	Lay health worker	Yes
	Anticipated regret	See examples below under “Complete TB evaluation.”	Lay health worker	Yes
	Information about social & environmental consequences	See examples below under “Complete TB evaluation.”	Lay health worker	Yes
	Comparative imaginings of future outcomes	See examples below under “Complete TB evaluation.”	Lay health worker	Yes
Modeling	Demonstration of the behavior	Invite former index TB patient to share the difficult decision to agree to household contact investigation.	Former TB patient	Worth considering
Enablement	Restructuring of the social environment	Seek permission for the home visit from the head of household by telephone instead of asking the index patients to consent.	Lay health worker	Yes, only as needed
Environmental restructuring	Restructuring of the physical environment	Screen the index patient by phone for greater privacy and convenience, if preferred.	Lay health worker	Yes, only as needed
**Screen contacts for TB (lay health workers)**				
Education	Instruction on performing the behavior	Provide a lecture about how to carry out TB screening.	TB Program	Yes
Training	Behavioral practice/rehearsal	Perform TB counseling role plays with one another.	Lay health worker	Yes
Persuasion	Framing/reframing	Describe the first priority of the home visit as supporting the index patient during treatment rather than as performing symptom screening.	Lay health worker	Yes
Enablement	Prompts/cues	Provide decision support on which contacts to refer for TB diagnostic evaluation using answers to questions about TB symptoms and predisposing factors.	mHealth / eTablet	Yes
Environmental restructuring	Adding objects to the environment	Provide lay health workers with N95 particulate respirators to reduce the risk and fear of contracting TB during household visits.	TB Program	Yes
	Restructuring the physical environment	Screen unavailable household contacts by phone for greater privacy and convenience if contacts prefer.	Lay health worker	Yes, only as needed
Incentivization	Material incentive (behavior)	Receive a modest allowance for transportation to the community and for meals.	TB Program	Yes
**Complete TB evaluation (contacts)**				
Education	Information about health consequences	Give positive/negative health information about health consequences of seeking/not seeking TB/HIV evaluation, treatment, and/or prevention.	Lay health worker	Yes
Persuasion	Credible source	Explain that index patient/contacts that TB health information has been approved by the leading TB authority in Uganda, the National TB Program.	Lay health worker	Yes
	Anticipated regret	Describe the regret that the index patient/contact could experience if screen-positive contacts do not receive evaluation & treatment.	Lay health worker	Yes
	Information about social & environmental consequences	Give positive/negative health information about social consequences of seeking/not seeking TB/HIV care, including putting other contacts at risk.	Lay health worker	Yes
	Comparative imaginings of future outcomes	Invite index patient/contacts to explicitly compare outcomes of screen-positive contacts receiving/not receiving TB/HIV evaluation/care.	Lay health worker	Yes
Environmental restructuring	Restructuring the physical environment	Collect sputum and provide HIV counseling and testing at home instead of in a clinic, using a safe and convenient place in or near the home.	Lay health worker	Yes, but randomize
		Deliver automated survey about TB symptoms every 6 months for 2 years for those found not to have TB and not treated for latent TB infection.	SMS	Yes, but randomize
	Restructuring the social environment	Provide TB and HIV testing at home, a less threatening social environment than the clinic.	Lay health worker	Yes, but randomize
Training	Instruction on performing the behavior	Instruct screen-positive contacts on how to expectorate sputum for TB examination safely and effectively at home.	Lay health worker	Yes, but randomize
Enablement	Action planning	Ask screen-positive contacts to schedule a time to go to clinic for TB/HIV evaluation.	Lay health worker	Yes
	Commitment	Ask screen-positive contacts to formally commit to going to clinic for TB/HIV evaluation.	Lay health worker	Yes
	Social support—emotional	Encourage screen-positive contacts invited to return to clinic together to provide mutual emotional support.	Lay health worker	Yes
	Feedback on outcome of behavior	Deliver results of sputum examination to contacts and recommend next steps.	Lay health worker, or Automated SMS	Yes
Incentivization	Non-specific reward	Arrange for screen-positive contacts to bypass the clinic waiting area and go directly to the TB unit when presenting for TB diagnostic evaluation.	Lay health worker	Yes
	Incentive (outcome)	Provide a small electronic cash transfer if a screen-positive contact returns to clinic for TB diagnostic evaluation.	SMS	No, not feasible

*SMS, short messaging services**;** TB, tuberculosis*.

*The table shows an implementation mapping exercise using the Behavior Change Wheel Framework and Behavior Change Techniques Taxonomy for each of the three key target behaviors (and the individual targeted). The intervention functions identified in Table 2 provide the starting point for Table 3, where candidate behavior change techniques are considered for each intervention function from a matrix listing all possibilities (36). The decision about whether to implement each of these behavior change techniques with their setting-specific content and mode of delivery was based on subjective ratings by implementers and stakeholders using the APEASE (Acceptability, Affordability, Practicality, Effectiveness/cost-effectiveness, Safety, and Equity) criteria, a subjectively assessed set of implementation and service outcomes*.

To change the second target behavior of lay health workers to enable them to screen more contacts for active TB, we identified multiple possible behavior change techniques, including (1) providing *instruction on performing the behavior* through lectures about how to carry out TB screening; (2) encouraging *behavioral practice/rehearsal* through role plays with one another; (3) *framing/reframing* the first priority of the home visit as supporting the index patient during treatment rather than as performing symptom screening; (5) providing electronic *prompts/cues* to lay health workers using decision support on electronic tablets to guide whom to refer to clinic for further evaluation; (6) *adding objects to the environment* by providing lay health workers with N95 respirators to reduce the risk and fear of contracting TB; and (7) *providing material incentives for the behavior* in the form of a modest financial allowance to lay health workers for transportation to the community and for meals. We also considered one other behavior change technique but did not select it routinely because it was not deemed feasible or acceptable to programmatic officials: (8) *restructuring the physical environment* by screening unavailable contacts by phone.

To change the third target behavior, getting household contacts to complete TB evaluation, we also identified multiple potential behavior change techniques. Several of these, including (1) *information about health consequences*, (2) *credible source*, (3) *anticipated regret*, (4) *information about social & environmental consequences*, and (5) *comparative imaginings of future outcomes* were selected with very similar content and modes of delivery as used for the first target behavior of encouraging index patients to agree to contact investigation. There were also several other possible behavior change techniques that we identified, including (6) *restructuring the physical environment* by collecting sputum and performing HIV counseling and testing at home, a more convenient and accessible location for testing than the clinic and by asking follow-up screening questions by SMS; (7) *restructuring the social environment*, by initiating TB and HIV testing at home, a less threatening social environment than the clinic; (8) providing *instruction on how to perform a behavior*, specifically sputum expectoration for TB examination; (9) encouraging *action planning*, by asking contacts to schedule a time to complete TB evaluation in clinic, (10) seeking a *commitment* in the form of a promise to complete TB evaluation in clinic; (11) recommending *emotional social support* by encouraging contacts to travel to clinic together; (12) providing *feedback on the outcome of the target behavior* by delivering results and follow-up instructions via SMS; and (13) offering a *non-specific reward* by enabling contacts to bypass the clinic waiting area when they present for TB evaluation. We also identified (14) providing an *incentive for the outcome* in the form of a small electronic cash transfer upon returning to the clinic, but did not include it, as it was not deemed feasible or acceptable by programmatic stakeholders. All selected behavior change techniques were integrated into contact investigation training materials, procedures, and operating protocols, for easy reference during the trial.

Finally, we also identified three policy changes that could leverage the impact of the selected intervention functions as part of an integrated implementation strategy. The first was a service delivery innovation, shifting responsibility for contact investigation from already over-burdened clinic heath care workers to lay health workers. The design team identified a large body of evidence supporting the feasibility, acceptability, and effectiveness of lay health workers in delivering community interventions for TB treatment and other disorders, when provided adequate training, supplies, and modest compensation (43). In addition, health care workers identified them as uniquely suited to this work. Second, a print and radio advertising campaign to increase general awareness of TB in the community and specific awareness of the new household contact investigation services was proposed and launched by a non-governmental organization serving as implementing partners to the National TB program in Kampala. Finally, local guidelines on contact investigation were envisioned, and these were developed by the National TB Program with input from the study team and other local experts and released in 2019 (44). Table 4 shows a logic model that summarizes the design of the implementation strategy to improve household TB contact investigation, highlighting the resources, activities, outputs, outcomes, and impact assessment plans (45).

**Table 4:** Logic model for design of a novel implementation strategy to adapt and deliver household TB contact investigation.

**Resources**	**Activities**	**Outputs**	**Outcomes**	**Impact assessment**
*Evidence & Guidelines*	*Reviewing evidence*	*Adapted evidence*	*Implementation protocol*	*Drafting evaluation protocol*
WHO	Document review	New TB diagnostic policies	Ethical approvals	Trial registration
TB-CARE	Attending CI training	CI implementation guide	Regulatory approvals	Design of fidelity studies
NTLP	Inviting local expert input	NTRL diagnostic guidelines		
Systematic reviews	Identifying gaps	New CI literature		
Targeted reviews	Projecting uptake	Cascade of CI delivery		
*Frontline stakeholders*	*Engaging & soliciting input*	*Summaries of input*	*Prepared stakeholders*	*Metrics for M&E*
Index TB patients	Direct observation	Key behaviors	Education & training	Feasibility measures
Household contacts	Focus group discussions	Key themes	Pilot testing	Acceptability measures
Clinic patients	Surveys	Behavioral determinants	Direct observation	Fidelity measures
Clinic workers	Process mapping	Targeted interventions	Data review	Outcome measures
Lay health workers	Skill assessments	Behavior change techniques	Protocol revision	
*Implementers*	*Building collaborations*	*Dialogue with implementers*	*Coordinated implementation*	*Disseminating results/plans*
Uganda MoH	Exchanging information	New TB diagnostic policies	Sharing preliminary results	Local presentations
Capital City Council	One-on-one meetings	Facility renovations	CI/adherence support bundle	Local reports
Research groups	Exchanging ideas	Kampala TB CI rollout	Troubleshooting technologies	Scientific publications
International NGOs	Coordinating roll-out	Staffing agreements		
Community NGOs	Negotiating staff allocation	Mobile app prototype		
ICT vendors	Bidding & specification	Uganda TB CI Guidelines		

*CI, contact investigation; ICT, Information & Communications Technology; M&E, monitoring and evaluation; MoH, Ministry of Health; NGOs, non-governmental organizations; NTLP, National Tuberculosis and Leprosy Programme; NTRL, National TB Reference Lab; TB, tuberculosis, WHO, World Health Organization*.

*The table shows the progression, from left to right, of the intervention adaptation and implementation design process, which was characterized by multi-level engagement with stakeholders in order to adapt the WHO recommended household TB contact investigation intervention to the local context and plan for implementation. We began with a formative phase (Resources, Activities, Outputs columns) in which we (1) identified key contact investigation behaviors and activities in collaboration with stakeholders; (2) employed mixed-methods data collection to explore key questions of interest; (3) applied an established theory of behavior change to identify barriers and facilitators of key contact investigation behaviors; and (4) tailored behavior change techniques into implementation strategies targeted to overcome barriers and enhance facilitators. We subsequently moved to a summative phase (Outcomes, Impact Assessment columns) where first piloted then adapted and evaluated the delivery of TB contact investigation, comparing a client-centered, mHealth-facilitated implementation strategy with a standard approach*.

### Implementation and Evaluation

Between July 2016 and July 2017, we introduced and evaluated a multi-component implementation strategy to improve uptake and completion of contact investigation. Lay health workers had previously completed Ministry of Health approved trainings on TB contact investigation (5 days) and household HIV testing (4 weeks), training on electronic-tablet based data entry and decision-support by a regional information technology consultant (5 days), and completed a 9-month pre-trial pilot period delivering standard TB contact investigation. Prior to the launch of the trial, they completed a 5-day refresher training covering the specific behavior change techniques and intervention functions that emerged from the BCW design process. Specifically, lay health workers completed didactic and practice sessions with the components targeting uptake, including all of the client-centered education, persuasion, and enablement techniques laid out in Table 3, and were encouraged to tailor their use of specific techniques (*e.g*., weekend visits, language related to framing of invitations) to the preferences of participants. Lay health workers also completed training on implementation components targeting health-workers, and were similarly instructed to apply all of the environmental restructuring, incentivization, and enablement techniques in all households. The implementation effectiveness of these strategies was therefore evaluated in comparison to historical controls. These trainings were jointly delivered by National TB Program implementing partners and research staff, who also provided longitudinal supportive supervision and regular data audits; these were the only two implementation components not derived from BCW and they were implemented because high quality data was required to ensure the integrity of the evaluation. In the pre-post implementation evaluation, uptake of contact investigation (*i.e.*, the first key behavior) among index patients improved markedly from 31 to 79% after introduction of the implementation strategy, while uptake among contacts (*i.e.*, the second key behavior) improved from 89 to 99%, relative to the pilot period (28).

Finally, we evaluated the implementation strategy components that were targeting completion of TB contact investigation (*i.e.*, the third key behavior), including home-initiated HIV-TB testing and follow-up text messaging, in a household cluster-randomized, controlled implementation trial involving 471 eligible index TB patients and 919 household contacts (22). In the standard of care arm, eligible contacts were referred to clinics for TB and HIV testing and clinical evaluation and did not receive automated text messages. By the end of the trial, we saw no improvement in the proportion of individuals who completed TB evaluation at 60 days (20 vs. 18%, difference 2.5%, 95% CI −6 to 11%, *p* = 0.57), and these proportions were similar to the proportion of 20% observed in the pilot study carried out prior to the implementation period. The negative trial results were primarily attributable to low fidelity delivery of the core implementation components. First, home sputum collection was successful in only 39% of eligible contacts; the reasons for failure included lay health workers not carrying enough sputum collection cups to the home visit, lay health workers being afraid of contracting TB while collecting or transporting sputum; and clients not understanding how to produce sputum and anticipating stigma if neighbors saw or overheard them in the act of expectorating (46). Second, automated text messages were sent out from the data server to only 58% of contacts because of a coding error. Furthermore, only 19% of eligible contacts ultimately received, opened, read, and remembered the messages, for a variety of reasons, including a reliance on shared phones, a lack of electricity to charge phones, weak cell-phone networks in some communities, and a preference for chat and social media applications over SMS (47). Finally, although home HIV testing was feasible and accurate (48), rates of acceptance were low, primarily because of fear of positive results and anticipated stigma with testing (49).

## Discussion

The design of effective implementation strategies is a critical aspect of implementation science that merits greater empirical study to help foster testing and development of best practices (46), especially in low- and middle-income countries. It has been hypothesized that applying a structured approach to designing and selecting implementation strategies may facilitate delivery of evidence-based practices, enhance service and quality, and improve individual and population health outcomes (47). Several influential articles have laid out the theory and practice of designing implementation strategies (30, 48, 49), but there have been relatively few examples of how these approaches can be applied in low-income countries. In addition, it is still unknown how best to tailor interventions to promote implementation (17). Here we have provided a comprehensive overview of our use of a leading implementation planning framework, the Behavior Change Wheel framework. Use of this framework enabled us to develop a multi-component implementation strategy to improve delivery of TB contact investigation, an evidence-based practice that has not been widely or effectively adopted in low-income countries.

When our multi-component strategy was prospectively evaluated, it was extremely successful at increasing uptake of contact investigation among both cases and contacts, but unsuccessful at improving completion of TB evaluation among eligible contacts. While both lay health workers and clients found the implementation components resulting from the theory-informed design process to be feasible and acceptable (50), the delivery of the two key implementation components, home sputum collection (51) and SMS messages (52) lacked fidelity leading to implementation failure. Our results were similar to those from two recent negative randomized trials of BCW-informed interventions, one delivering thrombolytic therapy for stroke in Australia (53) and the other promoting physical activity among adults at risk for cardiovascular disease in the Netherlands (54). Similar to our experience, the authors of these studies found the BCW framework to be feasible and useful for rigorously selecting and specifying implementation components, as have other investigators planning trials of novel BCW-informed strategies to promote smoking cessation in China (55), encourage physical activity among adolescent girls in Ireland (56), and reduce sedentary behaviors at work in England (57). The two groups that observed implementation failures, the Australian thrombolytic therapy group and the Dutch physical activity group, identified challenges with implementation fidelity and a compressed implementation period as factors that limited engagement of the health care workers whom their implementation strategies targeted. These findings contrast with two prior studies that found BCW-informed strategies to be effective for reducing inclusion of unhealthy foods in school lunches in Australia (58) and for preventing melioidosis in Thailand (59). A search of PubMed and clinicaltrials.org at the end of 2021 identified more than a dozen trials of BCW-informed implementation strategies that were planned, ongoing, or completed and awaiting publication, offering additional opportunities for evaluating the theory-informed design approach.

There were several strengths to our approach. First, we engaged stakeholders at multiple levels of the health system, from household contacts to the national TB program manager to international content experts in contact investigation. Second, we applied a systematic approach to identifying barriers to and facilitators of change, in which we defined the target behaviors of interest and collected extensive amounts of quantitative data to localize practice gaps and qualitative data about emergent themes that might help explain or mitigate these gaps. Finally, we applied a unifying theory of behavior change to develop a behavioral diagnosis for the practice gaps and a prescription for components of an implementation strategy targeted to overcome these gaps. Notably, we found the BCW approach to be equally applicable to both implementers and clients, demonstrating the flexibility of planned behavior change strategies across multiple levels of implementation.

There were also a few limitations to our approach. First, we only considered three general behaviors, a simplification that did not permit us to design for the micro-behaviors of sputum collection and text messaging that gave rise to the key implementation failures. Second, we did not include index patients in our initial qualitative studies, although we did directly observe their participation, survey them on their reasons for non-participation, and elicit information on their perspectives from household contacts and lay health workers (19, 28). Third, our approach, while comprehensive, produced a large number of potential behavior change techniques, too many for us to systematically evaluate for potential effectiveness. Preliminary evaluation of the individual implementation components might have allowed additional opportunities for iterative adaptation to improve the fidelity and fit of the strategy to the local setting (60, 61). Finally, we did not systematically assess organizational readiness (62), to identify individual and health system factors that might have facilitated adaptation at an earlier stage, although we did partner closely with programmatic leaders and implementing partners.

Beyond challenges with implementation fidelity that may or may not be attributable to the design process, we hypothesize that theory-informed design using the Behavior Change Wheel may have other limitations. First and most importantly, the Behavior Change Techniques Taxonomy includes only individual behavior change strategies, and the BCW framework does not offer specific methods for enacting change at the organization level, beyond a few general policies. In contrast, implementation mapping and the Expert Recommendations for Implementing Change (ERIC) approach offer methods for organizational change (16). Second, selecting appropriate intervention functions and behavior change techniques for producing strategies well-targeted to the underlying behavioral determinants, there may still be a need for additional tailoring of these implementation components to the local context. In this regard, showing that a strategy is acceptable may provide sufficient justification for a TB program to supply that service but may not actually increase demand for that service in a world where clients face choices and tradeoffs about if, when, and how to engage with implementers. While theory-informed design excels at identifying functions (referred to as “methods” in intervention mapping parlance), there is a need for greater attention to developing the forms of the implementation strategy (what intervention mapping calls “practical applications”) (38). Better forms may help ensure that the resulting implementation strategy truly suits the needs of end-users, and one way of achieving this is through iterative refinement prior to or during implementation. Future studies should therefore explore experimental and adaptive approaches to selecting and tailoring implementation components, including the multiphase optimization strategy (MOST) (63), and experiential and empirical methods like human-centered design (64). The ultimate goal should be to ensure that the most active implementation components can be refined to improve their feasibility, acceptability, and fit to the target setting and context.

## Data Availability Statement

The original contributions presented in the study are included in the article; further inquiries can be directed to the corresponding author.

## Ethics Statement

All studies involving human participants were reviewed and approved by the Uganda National Council for Science and Technology, the Makerere University School of Medicine Research Ethics Committee, the Committee on Human Research at the University of California San Francisco, and the Yale University Human Investigation Committee. Participants and/or their legal guardians/next of kin provided written informed consent and/or assent for contact investigation study activities. In addition, health workers provided either written or verbal consent for participation in qualitative studies.

## Author Contributions

Material preparation, data collection, and analysis were performed by JLD, IA, AJG, MA-H, JG, EO, DB, and PT. The first draft of the manuscript was written by JLD. All authors commented on previous versions of the manuscript, read and approved the final manuscript, and contributed to the study conception and design.

## Funding

This work was funded by NIH R01AI104824 (JLD), Nina Ireland Program in Lung Health, University of California, San Francisco (JLD), and NIH D43TW009607 (JLD).

## Conflict of Interest

The authors declare that the research was conducted in the absence of any commercial or financial relationships that could be construed as a potential conflict of interest.

## Publisher's Note

All claims expressed in this article are solely those of the authors and do not necessarily represent those of their affiliated organizations, or those of the publisher, the editors and the reviewers. Any product that may be evaluated in this article, or claim that may be made by its manufacturer, is not guaranteed or endorsed by the publisher.
